# Role of cytoskeletal proteins in cerebral cavernous malformation signaling pathways: a proteomic analysis†
†Electronic supplementary information (ESI) available. See DOI: 10.1039/c3mb70199a



**DOI:** 10.1039/c3mb70199a

**Published:** 2014-04-25

**Authors:** Sarah Schwartz Baxter, Christopher F. Dibble, Warren C. Byrd, Jim Carlson, Charles Russell Mack, Ivandario Saldarriaga, Sompop Bencharit

**Affiliations:** a David H. Murdock Research Institute , North Carolina Research Campus , Kannapolis , NC 28081 , USA; b Department of Pharmacology , School of Medicine , University of North Carolina , Chapel Hill , NC 27599 , USA; c Department of Prosthodontics and the Dental Research Center , School of Dentistry , University of North Carolina , Chapel Hill , NC 27599 , USA

## Abstract

Three genetic mutations were found to cause cerebral cavernous malformation (CCM), a vascular anomaly predisposing affected individuals to hemorrhagic stroke. These CCM proteins function together as a protein complex in the cell. Loss of expression of each CCM gene results in loss of *in vitro* endothelial tube formation. Label-free differential protein expression analysis using multidimensional liquid chromatography/tandem mass spectrometry (2D-LC-MS/MS) was applied to explore the proteomic profile for loss of each CCM gene expression in mouse endothelial stem cells (MEES) compared to mock shRNA and no shRNA control cell-lines. Differentially expressed proteins were identified (*p* < 0.05). 120 proteins were differentially expressed among the cell-lines. Principal component analysis and cluster analysis show the effects of individual knockdown. In all knockdown cell-lines, altered expression of cytoskeletal proteins is the most common. While all CCM mutations result in similar pathology, different CCM mutations have their own distinct pathogenesis in cell signaling.

## Abstract 2

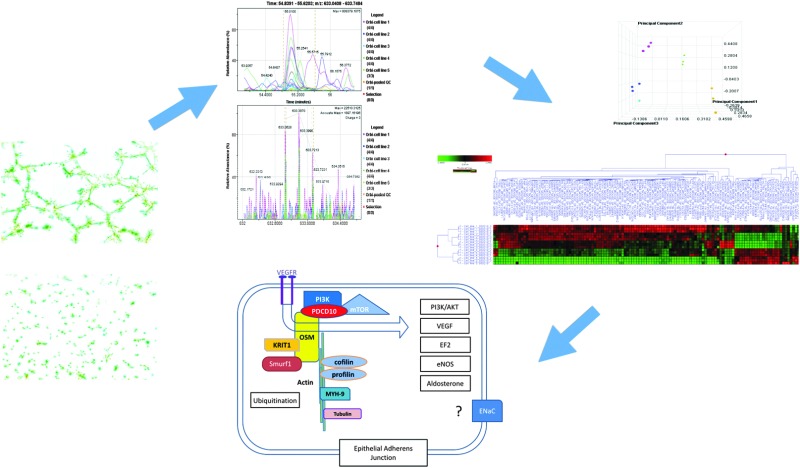
An *in vitro* proteomics and systems biology of cerebral cavernous malformation.

Cerebral cavernous malformations (CCM) are vascular disorders of the central nervous system (CNS) predisposing affected individuals to hemorrhagic stroke.^1,2^ Most CCM are detected by routine Magnetic Resonance Imaging (MRI) or at autopsy, with prevalence ranging from 0.1 to 0.5 percent in the general population. In the US, Hispanics may have a higher prevalence of CCM than do other ethnic groups, due to inherited mutations.^3^ Patients with a known CCM gene mutation carry two copies of the gene, one good copy and another mutated one. CCM lesions are often not detected until after the third or fourth decade of life.^1^ It therefore has been proposed that loss of the good copy of the gene may have caused the development of the lesion similar to precancerous cell development known as Knudsen hypothesis.^4^ In addition, mutations of a CCM gene can cause loss of protein expression or interaction of CCM proteins and their partners.^5^ Introducing a mutation or knockdown of a CCM gene therefore in theory may mimic the CCM lesion development in humans. Histopathologically, CCM are abnormally large hamartomatous vascular lesions formed by a single layer of capillary endothelial cells without the support of brain parenchyma.^1,2^ These lesions have a prospective hemorrhage rate of 3.1% per lesion per year. Patients with CCM may also have hemangiomas or CCM-like lesions in other organs including the spinal cord, skin, retina, liver, and vertebral column.^6^ Ruptured CCM lesions can cause hemorrhagic stroke and are often associated with seizures, recurrent headaches, and focal neurological defects.^1,7^ In patients with CCM lesions, each new lesion results in an increased seizure rate of 2.4% per year.^1,7^

Three CCM loci have been mapped in humans: 7q21-22 (CCM1), 7p13-15 (CCM2), and 3q25.2-27 (CCM3). Mutations in these CCM loci (*e.g.* frameshift, nonsense, splice-site, missense, and multi-exon deletions) cause loss of function of these proteins and result in CCM.^8–13^ The genes for CCM1, CCM2, and CCM3 encode Krit1 (krev interaction trapped 1), OSM (osmosensing scaffold for MEKK3) or malcaverin, and PDCD10 (programmed cell death 10), respectively. The mode of inheritance for congenital CCM is autosomal dominant. Although haplo-insufficiency is one model to explain the genetic basis of CCM, there is evidence that a Knudson-like two-hit mechanism may initiate lesion development.^14^

Krit1 or CCM1 protein was first described as a binder of krev-1 or rap1A, an evolutionary conserved Ras-family GTPase.^15–17^ Krit1 is composed of three domains, including an unstructured N-terminal domain containing three NPXY motifs, three ankyrin repeats (ANK), and a C-terminal Four-point one, Ezrin, Radixin, Moesin (FERM) domain. NPXY motifs are usually recognized by phosphotyrosine binding (PTB) domains and play an important role in protein–protein interactions.^3,18,19^ Both ankyrin repeats and FERM domains typically interact with actin.^18^ Krit1, a scaffold with no known catalytic activity, may function through its interaction with rap1A and the NPXY–PTB interactions with CCM2 protein (OSM) and Icap1 (integrin cytoplasmic domain associated protein). Icap1 is a 200 amino acid scaffolding protein encoding a PTB domain. Icap1 is known to bind small GTPases including Rac1 and Cdc42. Icap1 also interacts with the cytoplasmic domain of β1-integrin and participates in integrin-mediated signaling.^17–20^ The N-terminal portion of Krit1 also interacts with SNX17, a phosphatidylinositol-3,4,5-trisphosphate (PtdIns(3,4,5)P_3_) binding protein.^20^

OSM or CCM2 protein is composed of an N-terminal PTB domain (∼200 amino acids) and a C-terminal domain (∼300 amino acids) with no other recognizable structural motif. OSM was first described as a scaffolding protein that binds distinct kinases including MKK3 and MEKK3, as well as Rac1, and actin to coordinate the activation of the p38 MAPK cascade in response to hyperosmolarity.^21^ Our previous evolutionary studies show that the PTB domain of OSM has the closest relation to the PTB domain of Icap1.^22^ It is possible that OSM and Icap1 competitively interacts with the NPXY motifs in the N-terminal region of Krit1.^14,16–21,23^ OSM was first functionally identified as a scaffold protein that facilitates the activation of the MEKK3-MKK3-p38 MAPK cascade at sites of actin reorganization.^23^ OSM is localized, in part, to the actin cytoskeleton, and binds the small GTPase Rac1, the MAP3 kinase-MEKK3, and the MAP2 kinase-MKK3, an activator of p38. During osmotic stress, the MEKK3–OSM complex is recruited to membrane ruffles and both MEKK3 and the p38 stress-activated protein kinase are activated.^23,24^ A signaling complex of Krit1–OSM links the p38 activation of OSM with integrin-signaling through Krit1–Icap1 interaction.^24–26^ Additionally, we showed that the PTB domain of OSM binds the ubiquitin ligase (E3) Smurf1 which controls RhoA degradation and CCM2 knockdown phenotype can be recovered by overexpression of RhoA or inhibition of ROCK in endothelial cell culture.^27,28^

PDCD10 or CCM3 protein is a 25 KDa protein composed of only 212 amino acids. It was originally identified as TF-1 cell apoptosis related gene-15 (TFAR15), since it is up-regulated with the induction of apoptosis by serum withdrawal in TF-1 human premyeloid cells.^8,12^ It was subsequently renamed PDCD10 (programmed cell death 10 gene) as it was thought to be involved in apoptotic responses.^8,12^ PDCD10 is the third and the latest CCM gene identified.^8,12,29–31^ The N-terminal region of PDCD10, the site of L33-K50 deletion mutations in some CCM patients, was found to be the binding site for the oxidant stress response serine/threonine kinase 25 (STK25) and the mammalian Ste20-like kinase 4 (MST4).^26^ Similar to earlier observation of PDCD10, PDCD10 may function in apoptotic pathways since overexpression of wild-type PDCD10 induces apoptosis through caspase 3 pathway.^29^ PDCD10 may be regulated through phosphorylation and dephosphorylation, since it can be phosphorylated by STK25 and dephosphorylated by binding to the phosphatase domain of Fas-associated phosphatase-1.^26^ PDCD10 binds directly to OSM,^31^ and the PDCD10–OSM interaction is independent of the OSM–Krit1 interaction.^29,31^ We recently showed that PDCD10 binds specifically to PtdIns(3,4,5)P_3_ and the C-terminal amphipathic helix is responsible for PDCD10's function through its interaction with PtdIns(3,4,5)P_3_ and OSM. In addition, we previously proposed that PDCD10 may function in concerts with phosphoinositol-3 kinase (PI3K), the most important enzymes in the regulation of PtdIns(3,4,5)P_3_ at the plasma membrane.^31^

It is clear that all three CCM proteins function together and form complex in cell.^26,31^ In addition, individual knockdown of each CCM gene has been used as a tool to understand the function of each protein.^26,31^ However, while specific signaling pathways were revealed through studies of loss of CCM gene expression, the global proteomic changes resulting from these knockdowns are not known. Comparing the proteomic change resulting from individual CCM knockdown would reveal not only the common expressed proteins, but may also allow insight into novel signaling pathways related to each individual CCM gene. In this study, we examined the proteomic effects of loss of CCM gene expression in mouse endothelial embryonic stem cell (MEES) using label-free quantitative proteomic approach. Our analysis suggests that each CCM knockdown cell-line produces unique proteomic profiles corresponding to endothelial function phenotypes, and signaling proteins involved in cytoskeletal development are among the most common proteins in CCM knockdown cell-lines. The results suggest that proteomic analysis may be used as a tool to examine CCM related signaling pathways in future CCM studies.

## Results and discussion

### Global proteomic analysis

**Fig. 1:**
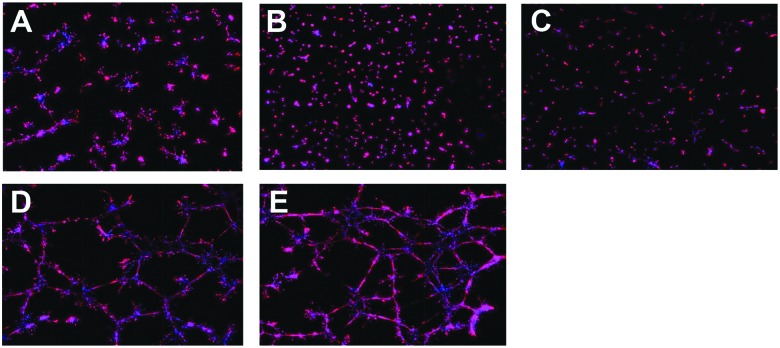
*In vitro* tube formation assays in MEES cell lines; (A) knockdown CCM1 (cell line 1), (B) knockdown CCM2 (cell line 2), (C) knockdown CCM2 (cell line 3), (D) wild-type (cell line 4), and (E) mock knockdown (cell line 5).

We compared each CCM knockdown cell line with a wild type and a mock knockdown to first look for proteins affected by the CCM knockdown. Then we compared the proteomic profiles among three CCM knockdowns. Expression of each of the CCM proteins in MEES cell lines was selectively inhibited by shRNA. A wild-type MEES cell line and a mock shRNA (pLKO.1-empty vector) cell line were used as controls. The levels of expression of each CCM gene after the knockdown were evaluated using Western analysis similar to our previous work.^31^ An *in vitro* tube formation assay was used to determine the functional deficiency of each of the cell lines after the knockdown (Fig. 1A–E). Note that all figures here use the following assignments: cell line 1 (knockdown CCM1), cell line 2 (knockdown CCM2), cell line 3 (knockdown CCM3), cell line 4 (wild-type MEES), and cell line 5 (mock knockdown). A knockdown of each CCM gene results in the inability to form endothelial tubes. This *in vitro* phenotype was believed to mimic the clinical CCM lesion in the brain.^31^ While the clinical and *in vitro* phenotypes look similar for all CCM mutations. Previous work from our group and others suggested that while there are certain common signaling pathways, there may be a unique pathway mediated by each individual CCM protein.

**Fig. 2:**
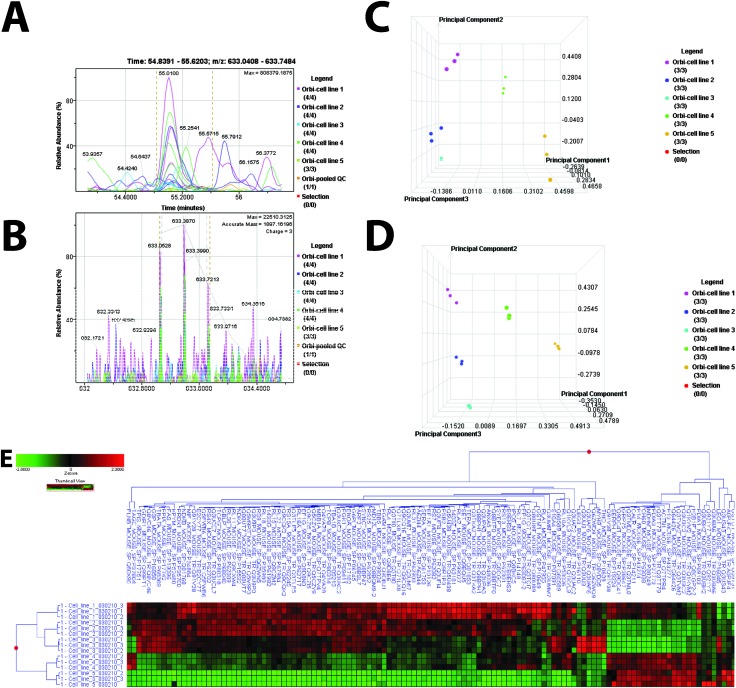
Mass Spectrometry Analysis (A–B). Aligned Mass Data. An example of aligned peptide signals for the data set is presented. Sample labels: figures are color coded according to cell line. Selection (red) references an option to manually select a single sample to be highlighted. This option was not selected. (A) Extracted ion chromatogram for *m*/*z* 633.0408–633.7484 from retention time 548 391–556 203 demonstrates retention time alignment across all samples for this signal. (B) Aligned masses in the mass range of 633.0408–633.7484 for all samples in the study set. (C). PCA plot for the study set. An ANOVA test was performed using all mass signals detected to compare the expression results from the five cell lines. Results demonstrate an unsupervised separation of the five cell lines based on the mass signal patterns. (D). PCA for cell line replicates. An ANOVA test was performed at the protein level to compare the protein expression results from the five cell lines. Candidate differentially expressed proteins were determined and are presented in the ESI,† Table S1. Results demonstrate separation of the five cell lines based on the protein patterns determined based on an ANOVA test. (E). Cell line cluster based on differentially expressed proteins determined by ANOVA.

To examine the global changes of each CCM knockdown, a label-free differential expression LC/MS/MS method was used to quantitatively compare the protein expression among all five cell lines; three CCM knockdown cell lines and two control cell lines (Fig. 1A–E). The total protein in each sample was calibrated (Fig. 2A and B). MS data from all five cell lines were analyzed in triplicate (15 replicates). Data processing in Elucidator was performed in multiple steps that included mass correction and mass signal alignment of detected signals, retention time alignment of detected signals, and feature selection, with features representing mass signals detected within the study set. QC review of the aligned data indicated the mass signals were correctly aligned in the Elucidator software (Fig. 2A and B). For retention time alignment, mathematical adjustments are performed to time-align the same detected signals across all samples. QC assessment of this retention time shift indicates that all adjustments were <3 min. Principal component analysis (PCA) was performed on the collected data set to evaluate the data for outliers (Fig. 2C and D) and sample trends. Results from the unsupervised analysis suggest that the samples differentiate by cell line based on the mass spectrometric patterns detected.

Comparing all five cell lines, the mass spectrometry analytical results were processed using the Rosetta Elucidator software. When applicable, mass signals were annotated with the corresponding peptide and protein information based on the database search results using a 1% false discovery rate cutoff. Data processing of the Thermo Orbitrap data resulted in detection of 177 590 features (mass signals). Of these, 3925 were annotated with peptide information that corresponded to the identification of 689 peptides corresponding to 258 proteins. PCA demonstrates a clear distinct proteomic profile for each cell line (Fig. 2C). We then compared all cell lines using an error-weighted ANOVA to determine mass patterns that correlated to differentially expressed proteins between the cell lines. Signals with a *p* < 0.01 were selected as tentative markers and summarized by protein. Total of 122 proteins are differentially expressed among three cell line samples. One hundred and ten proteins are differentially expressed among the three CCM knockdown cell lines. PCA again shows clear proteomic distinction of each cell line (Fig. 2D). Cluster heat maps based on these protein expression patterns, and the corresponding tentative differentially expressed signals are presented in Fig. 2E and ESI,† Table S1.

### Systems biology analysis

We further analyzed our proteomic data of each knockdown cell line compared with the mock knockdown using Ingenuity Pathways Analysis (IPA; Ingenuity Systems, California). Top five canonical signaling pathways common among the three comparisons are protein ubiquitination, EIF2 signaling, aldosterone signaling in epithelial cells, actin cytoskeleton signaling, and endothelial NO synthase (eNOS) signaling. We also noticed changes in proteins in vascular signaling pathways including PI3K/AKT signaling, VEGF signaling, mTOR signaling and RhoA signaling. Finally, signaling pathways involved in epithelial cell interaction, including remodeling of epithelial adherens junctions and epithelial adherens junction signaling are also noticed (Table 2 and ESI,† Table S2).

**Table 1:** Cytoskeletal proteins and proteins known to interact with CCM complex

Gene/sequence ID Protein description	Condition(s) with highest expression	Function	Subunits
EF1A1/P10126^*b*^ Elongation factor 1-alpha 1	Cell line 1, cell line 2	Enhances the binding of amino-acyl tRNA to the ribosome A-site, a GTP-dependent process	Binds to CCM protein complex

EF1G/Q9D8N0 Elongation factor 1-gamma	Cell line 1, cell line 2, cell line 3	Probably plays a role in anchoring the complex to other cellular components	EF-1 is composed of four subunits: alpha, beta, delta, and gamma

TCPB/P80314^*a*^ ^,^ ^*b*^ T-complex protein 1 subunit beta	Cell line 1, cell line 2, cell line 3	Serves as a molecular chaperone by assisting the folding of proteins upon ATP hydrolysis. Known to help in folding of actin and tubulin, *in vitro*	Exists as heterooligomeric complex (850 to 900 kDa) which forms two stacked rings

TCPG/P80318^*a*^ ^,^ ^*b*^ T-complex protein 1 subunit gamma	Cell line 1, cell line 2, cell line 3	Serves as a molecular chaperone by assisting the folding of proteins upon ATP hydrolysis. Known to help in folding of actin and tubulin, *in vitro*. Plays a part in the assembly of the von Hippel–Lindau ubiquitination complex	Exists as heterooligomeric complex (850 to 900 kDa) which forms two stacked rings

TAGL/P37804^*a*^ Transgelin	Cell line 1, cell line 2, cell line 4	Functions in actin cross-linking/gelling protein	

ENOA/P17182^*b*^ Enolase	Cell line 1, cell line 2, cell line 3	Multifunctional enzyme that, as well as its role in glycolysis, plays a part in various processes such as growth control, hypoxia tolerance and allergic responses. By similarity. May also function in the intravascular and pericellular fibrinolytic system due to its ability to serve as a receptor and activator of plasminogen on the cell surface of several cell-types such as leukocytes and neurons. Stimulates immunoglobulin production	Mammalian enolase is composed of 3 isozyme subunits, alpha, beta and gamma, which can form homodimers or heterodimers which are cell-type and development-specific. ENO1 interacts with PLG in the neuronal plasma membrane and promotes its activation. The C-terminal lysine is required for this binding by similarity. *In vitro*, interacts with several glycolytic enzymes including PKM2, PGM, CKM and aldolase. Also binds troponin, *in vitro*.

GBLP/P68040^*b*^ Guanine nucleotide-binding protein subunit beta-2-like 1	Cell line 1, cell line 2, cell line 3	Involved in the recruitment, assembly and/or regulation of a variety of signaling molecules. Interacts with a wide variety of proteins and plays a role in many cellular processes. Component of the 40S ribosomal subunit involved in translational repression. Binds to and stabilizes activated protein kinase C (PKC), increasing PKC-mediated phosphorylation. May recruit activated PKC to the ribosome, leading to phosphorylation of EIF6. Inhibits the activity of SRC kinases including SRC, LCK and YES1. Inhibits cell growth by prolonging the G0/G1 phase of the cell cycle. Enhances phosphorylation of BMAL1 by PRKCA and inhibits transcriptional activity of the BMAL1-CLOCK heterodimer. Facilitates ligand-independent nuclear translocation of AR following PKC activation, represses AR transactivation activity and is required for phosphorylation of AR by SRC. Modulates IGF1R-dependent integrin signaling and promotes cell spreading and contact with the extracellular matrix. Involved in PKC-dependent translocation of ADAM12 to the cell membrane. Promotes the ubiquitination and proteasome-mediated degradation of proteins such as CLEC1B and HIF1A. Required for VANGL2 membrane localization, inhibits Wnt signaling, and regulates cellular polarization and oriented cell division during gastrulation. Required for PTK2 phosphorylation and dephosphorylation. Regulates internalization of the muscarinic receptor CHRM2. Promotes apoptosis by increasing oligomerization of BAX and disrupting the interaction of BAX with the anti-apoptotic factor BCL2L. Inhibits TRPM6 channel activity. Regulates cell surface expression of some GPCRs such as TBXA2R. Plays a role in regulation of FLT1-mediated cell migration.	Component of the small (40S) ribosomal subunit. Exists as a monomer and also forms oligomers. Binds SLC9A3R1. Forms a ternary complex with TRIM63 and PRKCE. Interacts with HABP4, KRT1 and OTUB1. Interacts with SRC (*via* SH2 domain); the interaction is enhanced by tyrosine phosphorylation of GNB2L1/RACK1. Recruited in a circadian manner into a nuclear complex which also includes BMAL1 and PRKCA. Interacts with AR. Interacts with IGF1R but not with INSR. Interacts with ADAM12. Interacts with CLEC1B (*via* N-terminal region) and with HIF1A; the interaction promotes their degradation. Interacts with RHOA; this enhances RHOA activation and promotes cell migration. Interacts with CHRM2; the interaction regulates CHRM2 internalization. Interacts with TRPM6 (*via* kinase domain). Interacts with PTK2; required for PTK2 phosphorylation and dephosphorylation. Interacts with FLT1.

MYH9/Q8VDD5^*a*^ Myosin-9	Cell line 1, cell line 3	Plays a role in cytokinesis, cell shape, and specialized functions such as secretion and capping	Exists as a hexamer consisting of 2 heavy chain subunits (MHC), 2 alkali light chain subunits (MLC) and 2 regulatory light chain subunits (MLC-2)

TBA1A/P68369^*a*^ ^,^ ^*b*^ Tubulin alpha-1A chain	Cell line 1, cell line 2, cell line 3	Serves as the major constituent of microtubules. It binds two moles of GTP, one located on an exchangeable site on the beta chain and the other at a non-exchangeable site on the alpha chain	Exists as a dimer of alpha and beta chains

TBA1B/P05213^*a*^ ^,^ ^*b*^ Tubulin alpha-1B chain	Cell line 1, cell line 2, cell line 3	Serves as the major constituent of microtubules. It binds two moles of GTP, one located on an exchangeable site on the beta chain and the other at a non-exchangeable site on the alpha chain	Exists as a dimer of alpha and beta chains

TBB5/P99024^*a*^ ^,^ ^*b*^ Tubulin beta-5 chain	Cell line 1, cell line 2, cell line 3	Serves as the major constituent of microtubules. It binds two moles of GTP, one located on an exchangeable site on the beta chain and the other at a non-exchangeable site on the alpha chain	Exists as a dimer of alpha and beta chains

^*a*^Cytoskeletal proteins.

^*b*^Proteins associated with CCM protein complex (Hilder *et al.* 2007).

**Table 2:** Top canonical pathways

Top canonical pathways		
	*p*-value	Ratio
Protein ubiquitination pathways	3.48 × 10^–07^	7/64
ElF2 signaling	4.87 × 10^–07^	7/70
Aldosterone signaling in epithelial cells	1.12 × 10^–05^	6/71
Actin cytoskeleton signaling	2.14 × 10^–05^	7/126
eNOS signaling	4.11 × 10^–05^	6/87

### Cytoskeleton proteins involved in CCM

**Fig. 3:**
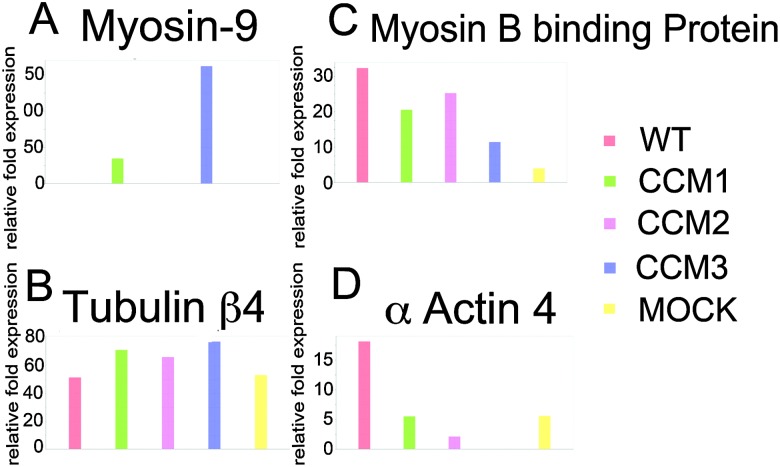
Differential expression of selected cytoskeletal proteins. Expression of cytoskeleton-related proteins based on relative fold expression (A) Myosin 9, (B) Tubulin β4, (C) Myosin B binding protein, and (D) α actin 4.

Our proteomic analysis shows that eight proteins differentially expressed among five cell lines are known proteins that interact with the CCM protein complex.^24^ Cytoskeletal proteins are among the most common (Table 1 and Fig. 3A–D). Both wild type (WT) and mock knockdown were used as a control. However, we compared the proteomic profiling of each knockdown with the mock knockdown to minimize confounding effects from the knockdown process. Gunel *et al.* ^32^ showed that Krit1, CCM1 protein, interacts with beta-tubulin. Béraud-Dufour *et al.* ^33^ further showed that Krit1-microtubule interaction is mediated with Rap1/Icap1. Rap1 and tubulin interact with Krit1's FERM domain, while Icap1 interacts through the N-terminal NPXY motif. The proteomic analysis shows a remarkable alteration of several tubulin isoforms. Our group previously showed that OSM, CCM2 protein, interacts with several isoforms of tubulin as well as elongation factor 1 alpha 1 (EF1A1). EF1A1 is one of the major regulators for actin cytoskeleton.^24^ This proteomic study shows the changes in EF1A1 and one of its partners-EF1G-in addition to direct interaction with cytoskeletal proteins, Krit1, OSM, and PDCD10 (CCM3 protein), may also regulate actin cytoskeleton through their interaction with phosphatidylinositol phosphates.^24,32,33^

**Fig. 4:**
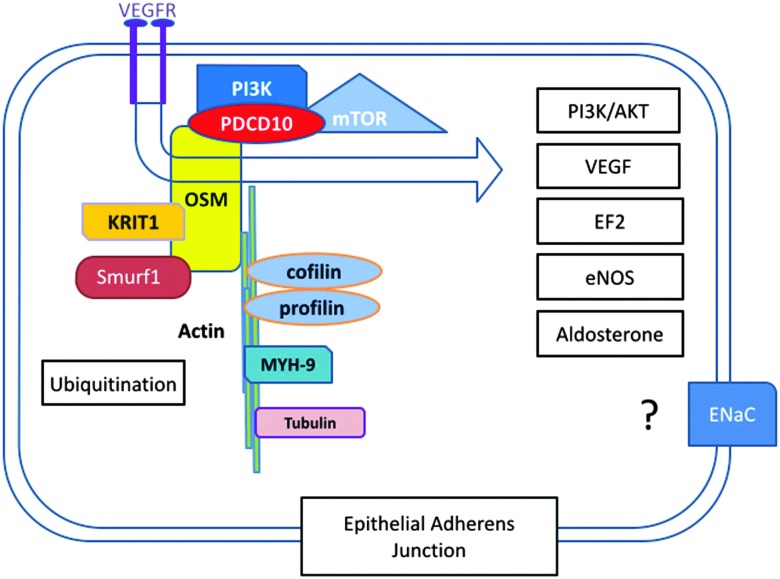
Summary model for signaling pathway for CCM development.

The proteomic alteration of cytoskeletal proteins, actin and tubulin isoforms, may be a result of loss of protein complex stability from missing CCM protein interaction. Microtubule stabilization through tubulin–protein interaction is known to regulate the cell polarization and proliferation. For instance, RASSF1A Tumor Suppressor is shown to be involved in the control of microtubule polymerization and in the maintenance of genomic stability.^27^ This may be similar to CCM complex stabilizing tubulin polymerization through their direct interaction. CCM proteins, *e.g.* Krit1 and OSM, are also known to interact with actin^17,23^ and, perhaps stabilize the actin–tubulin complex through their interactions with cofilin and profilin^34^ (Fig. 4). Cofilin, regulated by LIM-kinase 1,^34^ controls actin polymerization.^35^ Cofilin, known as actin-depolymerizing factors, functions with Arp2/3 complex in the opposite direction of profilin to control actin polymerization.^36,37^ Our proteomic results are inconclusive as to whether cofilin and profilin are differentially expressed among the knockdown, WT, and mock groups. (ESI,† Fig. S1) The relative fold expression of the groups for cofilin and profilin were included as a figure due to previous evidence for the proteins' involvement in the development of the CCM.^38^

### Translational control

In addition to controlling the cytoskeleton through direct interaction with actin and tubulin, CCM proteins may also regulate the expression of actin and tubulin through their interaction with transcriptional factors and may be a translational complex. OSM interacts with the transcriptional factors, *e.g.* EF1A1 and stabilizes the elongation factor complex. This, in turn allows appropriate transcription of actin.^24^ Our proteomic examination show also the alteration of EF1A1 and EF1G, EF1A1 partner. In addition, several riboproteins show alteration of their expression in all CCM knockdowns. We also see changes in Eukaryotic translation initiation factor 4A1 and asparaginyl-tRNA synthetase. These proteomic alterations suggest changes in translational control.

### Pathways involved in CCM

Our IPA systems biology analysis provides important additional insights into our proteomic analysis. Protein ubiquitination pathway, one of the top five identified pathways, is perhaps a result of CCM protein complex interaction with ubiquitin ligase. CCM2 protein-OSM is known to interact with ubiquitin ligase 3, Smurf1,^28^ and in turn regulate RhoA and actin. The common pathways of VEGF-PI3K/AKT-mTOR are shown in Fig. 4. Activation of VEGF related pathway through PI3K and mTOR may play an important role in CCM development. In addition the EF2 signaling, another top signaling pathway in our study, is also regulated through mTOR.^39^ Similarly eNOS signaling, also a top signaling pathway, is known to be regulated by the PI3K/AKT pathway.^40^ Another top signaling pathway, the aldosterone in epithelial cell pathway, again link PI3K/AKT to the epithelial sodium channel (ENaC).^41^ However, the role of aldosterone and ENaC in CCM development is not known. While we have introduced the possibility of PI3K/AKT pathway in CCM previously,^31^ more research is needed to determine how these related pathways are activated and lead to CCM clinical lesions.

## Conclusions

Our proteomic analysis shows cytoskeletal proteins involvement in CCM development through dysregulation of translation controls. Our *in vitro* study has a common limitation as a cell-based analysis. There may be more variations in humans. However, the results provide a clue for future analysis of candidate proteins in humans. This may provide a better understanding for CCM pathological process and treatments. Molecular detection of cytoskeletal protein changes may allow early CCM diagnosis. In addition, treatment for dysregulation of actin–tubulin in endothelial cells may be the key to future CCM therapy.

## Methods

### Cell lines and knockdown cell lines

Cell culture and RNAi-bEND.3 and MEEC cells were purchased from ATCC. Lentiviral gene-specific shRNAs in pLKO.1 backbone were obtained from the University of North Carolina-Chapel Hill Lenti-shRNA Core Facility. Lentiviral infection was performed according to the RNAi consortium protocol. bEND.3 cells were maintained in 10% FBS/DMEM and MEECs in 3% FBS/DMEM. 4 μg mL^–1^ puromycin was used to maintain shRNA selection.

### Mass spectrometry

Samples were analyzed using a simultaneous label-free differential protein expression approach. Samples were normalized to protein concentration, reduced, alkylated and trypsin digested. An alcohol dehydrogenase internal standard was added to the final digest, separated on a Waters nanoAcquity UPLC, and analyzed on a Thermo LTQ Orbitrap mass spectrometry system. MS data was processed using Rosetta Elucidator (Rosetta Biosoftware). Data processing steps included data pre-processing (mass calibration), mass signal alignment, protein database searching and identification using Mascot (Matrix Science) SwissProt mouse databases. Study results include data from 5 cell lines analyzed in triplicate (total of 15 replicates). Data processing in Elucidator was performed in multiple steps that included mass correction and mass signal alignment of detected signals, retention time alignment of detected signals, and feature selection, with features representing mass signals detected within the study set. QC review of the aligned data indicated the mass signals were correctly aligned in the Elucidator software (Fig. 2A and B). When applicable, mass signals were annotated with the corresponding peptide and protein information based on the database search results using a 1% false discovery rate cutoff. For retention time alignment, mathematical adjustments are performed to time-align the same detected signals across all samples. QC assessment of this retention time shift indicates that all adjustments were <3 min (data not shown). Principal component analysis (PCA) was performed on the collected data set to evaluate the data for outliers (Fig. 2C and D) and sample trends. Results from the unsupervised analysis suggest that the samples differentiate by cell line based on the mass spectrometric patterns detected. An error-weighted ANOVA was used to determine mass patterns that correlated to differentially expressed proteins between the cell lines. Signals with a *p* < 0.01 were selected as tentative markers and summarized by protein. PCA plots demonstrating replicate reproducibility and sample differences based on detected differentially expressed proteins are presented in Fig. 2C and D.

### Modeling using ingenuity pathway analysis

Ingenuity Pathways Analysis (IPA; Ingenuity Systems, California) was used to evaluate if any biological pathways or networks have any statistically significantly based on proteomic data of each CCM knockdown compared to the mock knockdown to determine the signaling pathways affected by each CCM gene knockdown. Protein accessions from each cell line (1 to 4; knockdown CCM1, 2, 3, and mock knockdown with *p* < 0.005) were imported into IPA. Focus proteins, mapped to corresponding gene objects in the Ingenuity Pathways Knowledgebase (IPKB), found in IPA were used to generate biological networks. IPA then built the networks with ∼120 proteins differentially expressed among cell-lines. For each network, *p*-value (indicating the likelihood of the focus proteins in a network being found together due to chance) and canonical pathway is calculated based on the fit of each set of significant proteins. This *p*-value is calculated from the right-tailed Fischer Exact Test. (; www.ingenuity.com) The IPA calculated the number of proteins in the pathway that meet cutoff criteria for differential expression *versus* the number that do not. This was used to generate a ratio of the pathways (Table 2).

## Author contributions

SSB and SB designed the experiments. SSB, JC performed mass spectrometry analysis. SB, SSB, JC, WCB, CRM, IS, and CFD analyzed data. CFP performed cell biology experiments and knockdowns. SB, WCB, and CRM wrote the manuscript.

## Conflicts of interest

The authors declare no financial conflicts of interest.
