# Supplementary material for: Role of cytoskeletal proteins in cerebral cavernous malformation signaling pathways: a proteomic analysis
Source: Mol Biosyst. 2014 Apr 25;10(7):1881–9. doi: 10.1039/c3mb70199a (PMC4043921; doi:10.1039/c3mb70199a)
Supplement: Supplementary file 1 [file MB-010-c3mb70199a-s001.pdf]

Electronic Supplementary Material (ESI) for Molecular BioSystems.
This journal is © The Royal Society of Chemistry 2014

Supplementary Table 1. Differentially Expressed Cell Line-Specific Proteins and Their Relative Levels of Expression.

	Cell line 4	Cell line 1	Cell line 2	Cell line 3	Cell line 5
**Identified Proteins (120)**	WT	ccm1	ccm2	ccm3	mock
**Putative uncharacterized protein** Q3TQ13	112.80	144.09	147.85	127.45	140.37
**Elongation factor** EF2	105.31	129.58	157.70	142.95	116.15
**MFLJ00343 protein (Fragment)** Q6KAM8	111.27	80.10	80.19	59.64	100.42
**Alpha-enolase** ENOA	79.14	108.11	110.29	110.52	96.69
**Putative uncharacterized protein** Q3UBU0	114.64	87.22	64.46	52.00	101.27
**Pyruvate kinase isozymes M1/M2** KPYM_MOUSE	85.43	90.44	95.65	94.76	88.10
**Putative uncharacterized protein** Q9DC41	112.67	86.21	64.19	64.56	107.20
**Heat shock protein HSP 90-alpha** HS90A	55.77	104.64	89.51	86.09	69.91
**Glyceraldehyde-3-phosphate dehydrogenase** D2KHZ9	72.54	73.36	93.79	84.45	78.65
**Transitional endoplasmic reticulum ATPase** TERA	58.80	51.88	53.26	44.56	84.34
**Elongation factor 1-alpha 1** EF1A1	65.50	81.40	78.87	79.18	77.10
**Clathrin, heavy polypeptide (Hc)** Q5SXR6	43.79	46.46	11.70	28.55	1.86
**Putative uncharacterized protein** Q3TIZ0	68.75	78.61	82.39	76.96	49.56
**Ahnak protein (Fragment)** A0PJF4	28.12	6.73	13.44	19.86	38.20
**Myosin-9** MYH9	1	34.52	0.00	161.97	1
**Heat shock protein 84b** Q71LX8	67.76	68.61	72.49	60.65	46.94
**Tubulin beta-4 chain** TBB4	51.55	71.32	66.14	77.35	53.27

	Cell line 4	Cell line 1	Cell line 2	Cell line 3	Cell line 5
**L-lactate dehydrogenase A chain** LDHA	57.30	64.33	66.19	64.65	40.31
**Putative uncharacterized protein** Q3UAA9	60.03	57.17	63.05	58.92	69.96
**Myb-binding protein 1A** MBB1A	32.28	20.52	25.18	11.41	3.97
**Putative uncharacterized protein** Q3UCD3	55.59	39.14	45.19	59.56	51.06
**Protein disulfide-isomerase A3** PDIA3	47.17	38.99	20.21	4.74	70.36
**Putative uncharacterized protein** Q8BV87	54.68	60.94	33.74	35.38	51.31
**Putative uncharacterized protein** Q8CE30	26.43	13.79	22.16	15.12	28.35
**Protein disulfide-isomerase** PDIA1	43.11	34.20	15.79	26.89	57.60
**Fructose-bisphosphate aldolase** A6ZI44	20.68	15.00	48.91	37.70	19.25
**Putative uncharacterized protein (Fragment)** Q3U7N9	24.46	3.37	12.72	12.34	31.21
**Putative uncharacterized protein** Q3UXC2	24.84	33.24	35.65	36.05	31.71
**Triosephosphate isomerase** TPIS	26.78	44.90	38.66	40.78	31.46
**Putative uncharacterized protein** Q3TVD2	41.23	27.32	23.02	11.31	39.25
**Putative uncharacterized protein** Q3U8U8	25.49	10.34	7.24	7.76	6.73
**Tln1 protein** Q0V930	10.01	4.60	2.06	2.89	28.49
**Protein disulfide-isomerase A4** PDIA4	26.43	7.71	1.01	0.96	27.54
**60 kDa heat shock protein, mitochondrial** CH60	32.47	24.36	1.05	0.00	52.67
**Adenylyl cyclase-associated protein** Q3TC53	35.28	30.86	32.79	25.86	38.74

	Cell line 4	Cell line 1	Cell line 2	Cell line 3	Cell line 5
**Non-muscle alpha-actinin 4** Q1A602	18.11	5.50	2.12	0.00	5.58
**Peroxiredoxin 1** B1AXW7	17.67	28.40	20.22	23.91	18.55
**Gag-pro-pol polyprotein** Q1KYL9	24.62	6.89	5.18	7.59	7.29
**Phosphoglycerate kinase 1** PGK1	9.79	11.24	20.12	27.53	10.10
**Putative uncharacterized protein** Q3TXW2	8.29	2.22	12.39	3.58	10.05
**Ubiquitin B** Q6NZC5	36.03	28.96	30.71	33.17	44.37
**Glucose-6-phosphate isomerase** G6PI	18.14	26.17	22.14	26.65	17.44
**Putative uncharacterized protein** Q8BGJ5	12.76	1.06	3.20	9.71	4.47
**Annexin A1** B7STB7	19.17	17.23	17.93	20.86	24.48
**Putative uncharacterized protein** Q564E8	24.71	28.96	30.67	21.59	19.65
**Tubulin beta-5 chain** TBB5	30.37	34.47	34.98	30.37	28.75
**Cofilin-1** COF1	26.49	27.65	33.88	20.95	31.81
**Ubiquitin-activating enzyme E1** B9EHN0	1.91	5.90	12.91	2.92	2.77
**Elongation factor 1-gamma** EF1G	17.01	16.08	13.54	17.94	12.16
**T-complex protein 1 subunit epsilon** TCPE	11.26	8.97	9.45	7.69	14.07
**D-3-phosphoglycerate dehydrogenase** SERA	19.08	18.54	24.29	13.23	7.99
**Phosphoglycerate mutase 1** PGAM1	23.65	31.35	27.45	26.62	25.98
**Putative uncharacterized protein** Q3U9Y8	30.46	25.35	10.64	9.55	2.81
**Putative uncharacterized protein** Q3UXQ6	18.11	24.13	32.75	25.73	14.63

	Cell line 4	Cell line 1	Cell line 2	Cell line 3	Cell line 5
**Ahnak protein (Fragment)** A0JLR7	11.64	3.45	10.57	19.83	14.73
**Ribosomal protein, large, P0** Q5FWB6	20.14	39.30	28.37	22.75	6.33
**6-phosphogluconate dehydrogenase, decarboxylating** 6PGD	2.03	14.94	16.73	25.70	10.15
**MKIAA0002 protein (Fragment)** Q6A0F1	4.22	0.00	10.37	9.22	0.00
**Putative uncharacterized protein** Q3TI62	15.36	25.13	24.16	22.58	15.98
**Protein disulfide-isomerase A6** PDIA6	20.74	18.30	11.68	6.66	26.18
**Annexin A5** ANXA5	17.17	13.71	19.07	18.07	17.04
**Putative uncharacterized protein** Q3U536	21.74	24.22	30.45	20.89	20.51
**Putative uncharacterized protein** Q3UB67	15.67	16.26	17.93	13.40	15.58
**Putative uncharacterized protein** Q542X7	8.32	5.65	13.79	6.70	9.15
**14-3-3 protein epsilon** 1433E	19.93	30.93	23.05	23.67	18.70
**Peptidyl-prolyl cis-trans isomerase** Q3UAJ1	23.46	16.10	27.52	20.79	22.37
**Transgelin-2** TAGL2	9.92	13.80	21.02	15.08	14.88
**T-complex protein 1 subunit delta** Q3TII0	8.51	8.05	13.73	8.49	3.82
**Putative uncharacterized protein** Q3UBI6	22.49	19.54	25.34	18.90	12.97
**Putative uncharacterized protein** Q3U6X2	14.04	17.49	15.91	10.41	15.68
**Putative uncharacterized protein** Q3UCL7	5.32	7.79	25.21	22.65	4.72
**40S ribosomal protein S7** RS7	24.08	19.76	26.25	18.97	22.67

	Cell line 4	Cell line 1	Cell line 2	Cell line 3	Cell line 5
**Guanine nucleotide-binding protein subunit beta-2-like 1** GBLP	14.39	13.77	22.20	16.08	0.85
**Down syndrome cell adhesion molecule-like protein (Fragment)** Q8R4B4	24.08	19.62	23.15	21.05	17.79
**Laminin receptor (Fragment)** B2CY77	13.29	24.36	17.93	14.19	15.98
**Putative uncharacterized protein** Q3UM23	3.81	0.00	0.00	21.03	3.77
**Putative uncharacterized protein** Q3UMM1	14.20	22.81	31.68	17.14	16.14
**Putative uncharacterized protein** Q3TXS9	13.33	19.53	22.10	18.90	4.73
**Putative uncharacterized protein** Q3TFK4	14.08	0.00	9.45	10.05	13.12
**Profilin** Q8CEH8	19.27	20.44	16.92	19.83	16.89
**Putative uncharacterized protein** Q8C483	14.26	5.76	7.34	15.09	2.71
**Identified yet undeclared protein #25315 in ProteinLynx output.** FAKE_25315	0.00	5.83	5.28	5.76	10.96
**Galectin-1** LEG1	16.96	14.84	12.65	20.86	17.84
**Alcohol dehydrogenase 1** ADH1	8.51	3.45	9.36	5.74	8.35
**Putative uncharacterized protein** Q3TJ69	9.70	2.29	12.74	23.67	9.60
**Importin-5** IPO5	0.84	2.31	0.00	7.36	1
**Peroxiredoxin-6** PRDX6	5.00	8.37	15.75	11.24	10.40
**Putative uncharacterized protein** Q9D1S3	7.13	9.19	12.52	7.46	1.71
**14-3-3 protein gamma** 1433G	10.54	21.58	19.96	16.97	6.43
**Heat shock protein 1-like protein** B2CSK2	3.94	6.91	11.44	15.28	4.92

	Cell line 4	Cell line 1	Cell line 2	Cell line 3	Cell line 5
**60S ribosomal protein L10**<br>RL10	2.53	15.84	16.82	15.98	7.54
**Inducible heat shock protein 70**<br>A1E2B8	19.95	12.78	9.58	9.42	10.56
**GTP-binding nuclear protein Ran**<br>RAN	8.94	8.03	12.65	15.42	6.58
**Poly(rC)-binding protein 1**<br>PCBP1	7.54	4.35	4.17	5.67	10.45
**S-formylglutathione hydrolase**<br>ESTD	0.84	2.31	3.26	13.20	11.11
**Malate dehydrogenase, mitochondrial**<br>MDHM	23.65	18.30	1.11	1	23.52
**Nucleosome assembly protein 1-like 1**<br>NP1L1	8.54	10.01	13.72	10.48	16.89
**Enolase**<br>Q4FK59	12.79	19.96	16.08	14.29	14.52
**60S ribosomal protein L6**<br>Q3UCH0	10.41	11.24	16.82	7.46	4.67
**Putative uncharacterized protein**<br>Q3U122	21.30	14.79	11.68	11.37	16.68
**Eukaryotic translation initiation factor 5A-1**<br>IF5A1	7.66	14.84	6.33	13.06	10.10
**Putative uncharacterized protein**<br>Q5BLJ7	11.29	18.39	14.80	14.19	10.40
**60S acidic ribosomal protein P2**<br>RLA2	11.42	12.54	18.75	13.20	15.93
**Putative uncharacterized protein**<br>Q58ET1	3.63	17.14	3.10	0.00	1
**Identified yet undeclared protein #23114 in ProteinLynx output.**<br>FAKE_23114	3.50	16.10	13.76	29.38	7.44
**Glutathione S-transferase Mu 1**<br>GSTM1	6.82	15.92	7.28	12.30	6.48
**Putative uncharacterized protein**<br>Q58EU6	9.07	0.00	5.26	1.86	6.48
**ATP synthase subunit beta, mitochondrial**<br>ATPB	7.16	0.00	1	1	27.24

	Cell line 4	Cell line 1	Cell line 2	Cell line 3	Cell line 5
**Heat shock protein 2**<br>Q99KD7	11.51	7.94	7.34	13.30	8.79
**Rps16 protein**<br>Q5CZY9	7.66	12.56	14.61	10.47	4.88
**Histone H2B**<br>A2AB77	14.39	9.19	6.23	2.95	22.57
**Histone H2A.x**<br>H2AX	20.99	18.37	3.14	1	20.81
**Enolase**<br>Q3UJ20	11.70	12.59	5.34	9.29	11.26
**Aldehyde dehydrogenase, mitochondrial**<br>ALDH2	0.00	1	1	1	17.74
**MC3T3-E1 calcyclin**<br>Q545I9	6.26	8.12	0.00	7.56	9.35
**Putative uncharacterized protein**<br>Q9CWK0	9.60	12.72	11.55	10.57	7.44
**Putative uncharacterized protein (Fragment)**<br>Q3U8X6	3.63	20.61	6.17	9.44	8.19
**Protein S100-A11**<br>S10AB	2.79	7.06	8.29	5.50	12.97
**Putative uncharacterized protein**<br>Q3UKW2	1.06	2.46	0.00	1.89	12.06
**Glutathione S-transferase Mu 2**<br>GSTM2	0.00	12.54	0.00	0.90	1
