# Supplementary material for: Role of cytoskeletal proteins in cerebral cavernous malformation signaling pathways: a proteomic analysis
Source: Mol Biosyst. 2014 Apr 25;10(7):1881–9. doi: 10.1039/c3mb70199a (PMC4043921; doi:10.1039/c3mb70199a)
Supplement: Supplementary file 2 [file MB-010-c3mb70199a-s002.pdf]

Electronic Supplementary Material (ESI) for Molecular BioSystems.
This journal is © The Royal Society of Chemistry 2014

Supplementary Table 2 - Top Molecules for Fold Change from Pathway Analysis (CCM1, CCM2, CCM3)			
**CCM1**			
**Upregulated**	Exp. Value	**Downregulated**	Exp. Value
**MYH9**	34.520	**PTBP1**	-12.038
**GSTM1**	15.675	**FLNA***	-7.258
**ESD**	2.750	**Serpinb9b**	-4.236
**S100A11**	2.530	**ACTN4**	-3.293
**GSTM5**	2.334	**PABPC1**	-2.465
**Calm1 (includes others)**	2.321	**TLN1**	-2.176
**YWHAG**	2.047	**NCL**	-1.917
**EIF5A**	1.937	**ACTA2**	-1.440
**HSP90AA1**	1.876	**ANXA2**	-1.420
**PRDX6**	1.674	**HSPD1**	-1.333
**CCM2**			
**Upregulated**	Exp. Value	**Downregulated**	Exp. Value
**ESD**	3.881	**HSPD1**	-30.924
**PRDX6**	3.150	**ACTN4**	-8.542
**S100A11**	2.971	**S100A6**	-7.825
**TAGLN2**	2.119	**TLN1**	-4.859
**PGK1**	2.055	**PTBP1**	-3.987
**RPS16**	1.907	**PABPC1**	-3.521
**YWHAG**	1.894	**ENO2**	-2.191
**RPS3A**	1.756	**FLNA***	-1.923
**RPS2**	1.658	**ACTA2**	-1.824
**HSP90AA1**	1.605	**HSP90B1**	-1.778
**CCM3**			

**Supplementary Table 2 - Top Molecules for Fold Change from Pathway Analysis (CCM1, CCM2, CCM3)**			
**Upregulated**	Exp. Value	**Downregulated**	Exp. Value
**MYH9**	161.970	**HSPD1**	-40.587
**ESD**	15.714	**ACTN4**	-22.638
**PGK1**	2.812	**TLN1**	-3.464
**Serpinb9b**	2.440	**PABPC1**	-3.285
**PRDX6**	2.248	**HSP90B1**	-2.205
**S10A11**	1.971	**FLNA***	-1.982
**GSTM5**	1.804	**ACTA2**	-1.873
**Calm1 (includes others)**	1.783	**NCL**	-1.748
**EIF5A**	1.705	**HSPA5**	-1.745
**YWHAG**	1.610	**SERPINH1**	-1.546
