# Supplementary figures and images for: Role of cytoskeletal proteins in cerebral cavernous malformation signaling pathways: a proteomic analysis
Source: Mol Biosyst. 2014 Apr 25;10(7):1881–9. doi: 10.1039/c3mb70199a (PMC4043921; doi:10.1039/c3mb70199a)

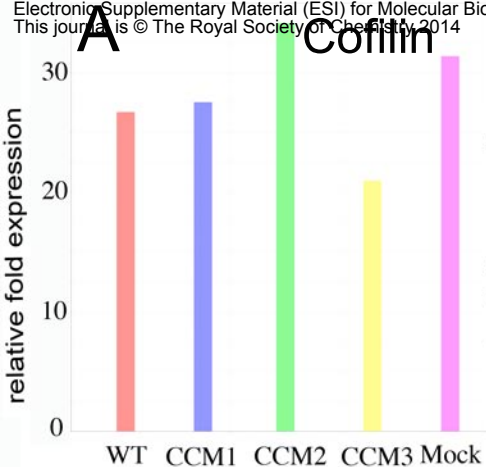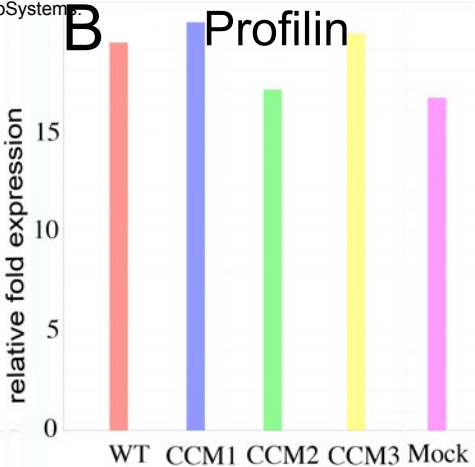

Supplement: Supplementary file 3 [file MB-010-c3mb70199a-s003.pdf]
